# Effectiveness and Safety of Abrocitinib in Patients with Moderate-to-Severe Atopic Dermatitis: A Systematic Review and Meta-Analysis of Randomized Clinical Trials

**DOI:** 10.1155/2021/8382761

**Published:** 2021-06-23

**Authors:** Hammad Ali Fadlalmola, Muayad Saud Albadrani, Amal Mohamed Elhusein, Wahieba E. Mohamedsalih, D. S. Veerabhadra Swamy, Daniel Mon Mamanao

**Affiliations:** ^1^Nursing College, Taibah University, Medina, Saudi Arabia; ^2^Medicine College, Taibah University, Medina, Saudi Arabia; ^3^College of Applied Medical Science, Bisha University, Bisha, Saudi Arabia; ^4^College of Nursing, Khartoum University, Khartoum, Sudan

## Abstract

**Background:**

Atopic dermatitis (AD) is a complex, chronic, inflammatory skin disease characterized by pruritic, intense itching, and eczematous lesions affecting about 25% of children and 2% to 3% of adults worldwide. Abrocitinib is a selective inhibitor of Janus kinase-1 (JAK1) enzyme inhibiting the inflammatory process. Therefore, we aimed to assess the efficacy and safety of abrocitinib for moderate-to-severe AD.

**Methods:**

We systematically searched PubMed, Cochrane, Web of Science, Scopus, and EczemATrials till Feb 1, 2021, for reliable trials. The analysis was conducted using an inverse-variance method. The results were pooled as mean difference/event rate and 95% confidence interval.

**Results:**

Abrocitinib 100 mg and 200 mg were associated with higher IGA response, EASI-50% responders, EASI-75% responders, EASI-90% responders, number of participants with at least 4-point improvements in NRS, and quality of life measured by DLQI and CDLQI than placebo. Also, 100 mg and 200 mg were associated with lower SCORAD index, %BSA, PSAAD index, and POEM index than placebo. Abrocitinib 100 mg and 200 mg were not associated with adverse events such as upper respiratory tract infection, nasopharyngitis, dermatitis, atopic, any serious adverse events, and death.

**Conclusion:**

Abrocitinib in dose 100 mg or 200 mg is an effective, well-tolerated, and promising drug in treating patients with moderate-to-severe atopic dermatitis. However, the analysis favored the efficacy of abrocitinib 200 mg over 100 mg, but side effects such as nausea and headache are likely to occur more with 200 mg.

## 1. Introduction

Atopic dermatitis (AD) is a complex, chronic, inflammatory skin disease characterized by pruritic, intense itching, and eczematous lesions. It is the most prevalent inflammatory dermal diseases, affecting 3–10% of adults and 15–25% of children in the USA [1] and up to 25% of children and 2% to 3% of adults worldwide [2]. Therefore, AD is frequently repeated in the clinical practice [3]. The origin of AD is multifocal, including mainly encountered triggers due to skin barrier dysfunction that leads to enhanced skin irritability to nonspecific stimuli and epicutaneous sensitization [3, 4]. The primary risk factors for atopic dermatitis are the family history of eczema, allergies, hay fever or asthma, and the first year of life exposure to high levels of ambient nitrogen dioxide (NO_2_) [5]. It is relapsing in nature, often with repeated flares, and may negatively impact the quality of life (QoL) for patients and their family members [5, 6].

There is no specific first-line medication for AD till now; however, some new medications are under investigation. The most frequent used medications for AD are symptomatic medications including emollients (moisturizers) used every day to prevent the skin from becoming dry, topical corticosteroids to reduce swelling and redness during flare-ups, antihistamines to reduce severe itching [7, 8]. Hence, additional treatments are needed for patients with moderate-to-severe AD.

Recently, there were other family medications for treating some immune-related condition and AD: Janus kinase-1 inhibitor (JAK) inhibitors. JAK inhibitors belong to a family of medicines called DMARDs (disease-modifying antirheumatic drugs). FDA approves three JAK inhibitors: baricitinib, tofacitinib, and upadacitinib, for treating rheumatoid arthritis [9]. Pharmacologically, JAK inhibitors are small molecular synthetic compounds inhibiting the intracellular signal transduction of cytokine receptors [10]. Cytokines are mediators in numerous inflammatory skin disorder. These cytokines, especially interleukin I and II, are involved in the process of skin damage in AD. JAK inhibitors work by modifying the immune system via inhibiting cytokine receptors by phosphorylation, decreasing the immune response, and improving AD symptoms [11]. JAK inhibitors are under clinical investigation for inflammatory skin diseases, specifically phase 3 trials for AD or psoriasis. As JAK inhibitors are tested in oral and topical formulations, they could become prevalent in dermal therapy [12].

Abrocitinib is a selective inhibitor of JAK1 enzyme [13]. JAK1 is a human tyrosine kinase protein important for signaling certain types of cytokines (type I and type II) and interacts with the common gamma chain (*γ*c) of type I cytokine receptors to elicit signals from the interleukin-2 (IL-2) receptor family initiating responses to multiple major cytokine receptor families [13, 14].

As JAK-1 inhibitors are a new promising drug family for treating AD and due to the lack of evidence regarding the new drug called abrocitinib, although there are very few systematic reviews that talk about abrocitinib and atopic dermatitis, this systematic review examined several outcomes and different doses 100 and 200 mg of treatment, resulting in a good evidence that may serve as a good reference to atopic dermatitis guidelines. In this systematic review and meta-analysis, we aimed to synthesize evidence regarding the efficacy and safety of abrocitinib 100 mg and 200 mg compared to placebo for patients suffering from AD.

## 2. Methods

### 2.1. Search Strategy and Data Collection

We searched five electronic databases, PubMed, Cochrane CENTRAL, Web of Science, Scopus, and Global Resource for EczemATrials (GREAT) (Centre of Evidence-Based Dermatology; http://www.greatdatabase.org.uk), for all published clinical trials till Feb 1^st^, 2021. Also, we searched for any published results from ongoing studies on the ongoing trials registry of the US National Institutes of Health (http://www.clinicaltrials.gov). The search was conducted using the following search strategy: (Abrocitinib OR Janus kinase 1 inhibitor OR JAK1 inhibitor OR PF-04965842) AND (Atopic dermatitis OR Atopic Dermatitides OR Atopic Neurodermatitides OR Atopic Neurodermatitis OR Disseminated Neurodermatitides OR Disseminated Neurodermatitis OR Neurodermatitis Disseminata OR Atopic Eczema OR Infantile Eczema OR flexural eczema OR prurigo Besnier OR allergic eczema OR Eczema Pruriginosum Allergicum).

Using Endnote software, we removed the duplicates; then, we screened all retrieved citations for eligibility through two steps: title and abstracts, then full text, and those matching our criteria were included in our study. We also screened the references of the included studies manually for additional relevant papers.

### 2.2. Selection Criteria

We included all randomized clinical trials (RCTs) that enrolled patients with moderate-to-severe atopic dermatitis and investigated abrocitinib's safety or efficacy in any dose compared to placebo. If more than one trial reported the same population, we used the most complete dataset of results. No restrictions for age, sex, site, or publication date were applied.

We excluded animal studies, observational studies, non-English studies, nonavailable studies, thesis reviews, and if the abstract only is available.

### 2.3. Data Extraction

We extracted data related to the following: (1) summary of the included trials including: study time and sites, design and phase, protocol NCT number, total number of patients, inclusion criteria, study arms and number of patients in each, dose, route, and regimen of abrocitinib, duration of treatment, AD severity, study conclusion, (2) baseline characteristics of the enrolled population including: age, sex, race, disease duration, Investigator's Global Assessment (IGA) grade, Eczema Area and Severity Index (EASI) score, % body surface area (BSA) affected, pruritus numeric rating scale (NRS) score, Scoring Atopic Dermatitis (SCORAD), Pruritus and Symptoms Assessment for Atopic Dermatitis (PSAAD), Patient Oriented Eczema Measure (POEM), Dermatology Life Quality Index (DLQI), Children's Dermatology Life Quality Index (CDLQI), previous medications for AD, (3) efficacy and safety outcomes, and (4) quality assessment domains.

### 2.4. Study Outcomes

We assessed the efficacy of abrocitinib in patients with AD through the following outcomes: IGA response, EASI 50%, 75%, 90% responders, participants with at least four points improvements in NRS, SCORAD Index, %BSA, PSAAD index, POEM index, quality of life by DLQI and CDQLI. Extracted safety measures included: death, serious adverse events, nausea, headache, dermatitis, atopic, nasopharyngitis, upper respiratory tract infection.

### 2.5. Quality Assessment

We assessed the quality of the included RCTs using Cochrane's risk of bias tool (version 1). The tool is found in chapter 8.5 of the Cochrane handbook of systematic reviews of interventions 5.1.0 [15]. The tool consists of the following domains: sequence generation (selection bias), allocation sequence concealment (selection bias), blinding of participants and personnel (performance bias), blinding of outcome assessors (detection bias), incomplete outcome data (attrition bias), selective outcome reporting (reporting bias), and other bias; author judgments fall into three categories: low, unclear, or high risk of bias for each domain.

We could not assess the risk of publication bias due to the small number of included studies, according to Egger's funnel-plot-based method [16].

### 2.6. Statistical Analysis

Continuous data were pooled as mean differences (MD) and 95% confidence intervals (CI) using the inverse-variance method, while dichotomous data were pooled as risk ratio (RR) and 95% CI using the Mantel–Haenszel method. We used the fixed-effect model when the pooled data are homogenous; otherwise, we used the random-effects model.

We used the Review Manager Software, version 5.3, to conduct the analysis. When the mean or standard deviation data are missing, we calculated their 95% CI according to Altman's equation [17].

## 3. Results

### 3.1. Literature Search Results

**Figure 1:**
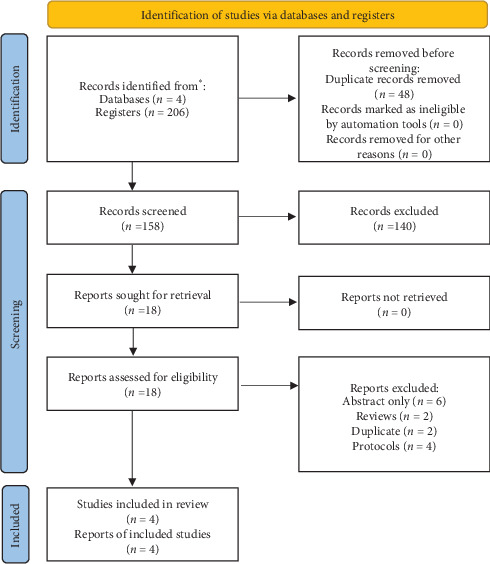
PRISMA flow diagram. It summarizes the results of searching databases and screening the obtained records.

Our search retrieved 158 citations after removing duplications, 140 records were excluded by title and abstract screening, and the remaining 18 were eligible for full-text screening. We finally included four trials in our study [13, 18–20]. The flow of data collection and screening process are shown in (Figure 1).

### 3.2. Summary of the Included Studies

The included trials compared between different drug doses (10, 30, 100, 200 mg) and placebo with a total sample size of 1882 patients. All patients had moderate-to-severe AD and received the drug or placebo orally, once daily for nearly 12 weeks. Mean age of included patients ranged from 31 to 45 years with at least 20 years disease duration. Summary of the included trials and baseline characteristics of enrolled subjects is shown in Tables 1 and 2, respectively.

### 3.3. Quality Assessment

**Figure 2:**
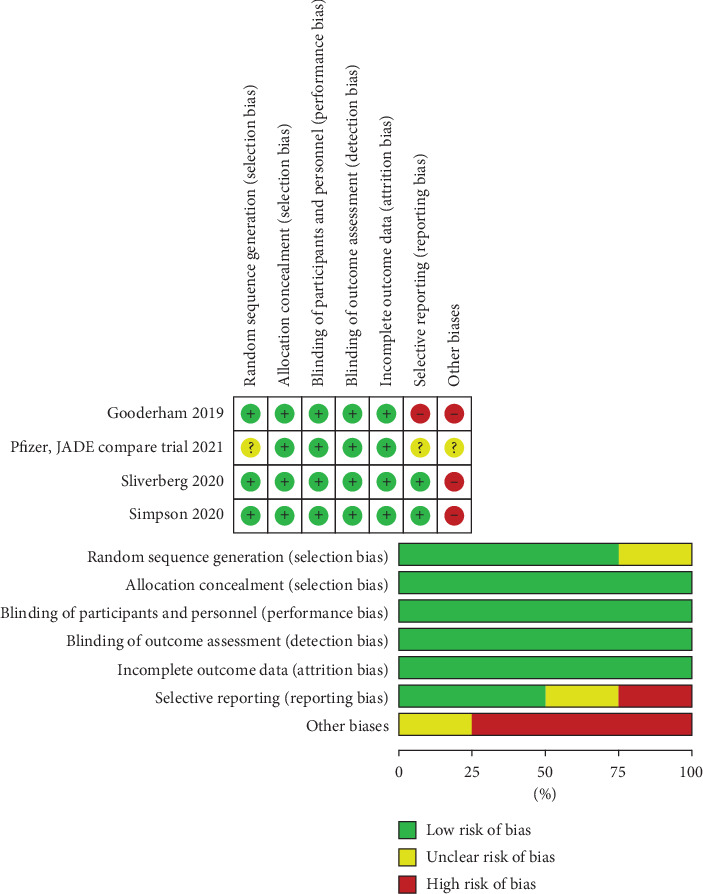
Risk of bias graph and summary.

The risk of bias assessment revealed that the included studies were high quality according to the Cochrane risk of bias tool. All studies were at low risk of bias regarding selection, except one, detection attrition performance bias. One trial [19] was unclear the risk of selection bias. Regarding reporting bias, two trials were low risk [18, 20], one trial was unclear [19], and the remaining was high risk [13]. Three studies [13, 18, 20] showed other sources of bias and the remaining was unclear [19]. Risk of bias graph and summary are shown in Figure 2.

## 4. Outcomes

### 4.1. IGA Response

**Figure 3:**
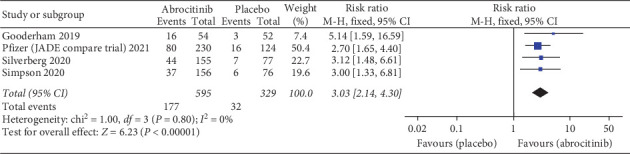
Forest plot of the IGA response (100 mg abrocitinib vs. placebo).

Pooled analysis revealed that 100 and 200 mg abrocitinib significantly increased IGA response more than placebo (RR = 3.03; 95% CI: [2.14, 4.30], *P* < 0.0001) (Figure 3) (RR = 4.44; 95% CI: [3.16, 6.24], *P* < 0.0001) (Supplementary Figure S1), respectively. Also, 200 mg abrocitinib was associated with higher IGA response more than 100 mg (RR = 1.47; 95% CI: [1.26, 1.72], *P* < 0.0001) (Supplementary Figure S2). Pooled results were homogenous (*I*^2^ = 0%, *P*=0.80) (*I*^2^ = 0%, *P*=0.62) (*I*^2^ = 0%, *P*=0.47), respectively.

### 4.2. EASI-50 Responders

Pooled analysis revealed that 100 and 200 mg abrocitinib significantly increased EASI-50 responders more than placebo (RR = 2.22; 95% CI: [1.38, 3.58], *P*=0.001) (RR = 2.83; 95% CI: [1.70, 4.72], *P* < 0.00001), respectively. Also, 200 mg abrocitinib was associated with higher EASI-50 responders more than 100 mg (RR = 1.23; 95% CI: [1.15, 1.32], *P* < 0.00001). Pooled results were heterogenous in 100 mg and 200 mg vs. placebo (*I*^2^ = 84%, *P*=0.0002) (*I*^2^ = 88%, *P* < 0.0001) and homogenous in 100 vs. 200 mg (*I*^2^ = 0%, *P*=0.44), respectively, and the heterogeneity was best resolved by excluding Pfizer (JADE compare trail) 2021 without effect on the significance.

### 4.3. EASI-75 Responders

Pooled analysis revealed that 100 and 200 mg abrocitinib significantly increased EASI-75 responders more than placebo (RR = 2.74; 95% CI: [1.99, 3.79], *P* < 0.00001) (RR = 4.04; 95% CI: [2.55, 6.42], *P* < 0.00001), respectively. Also, 200 mg abrocitinib was associated with higher EASI-75 responders more than 100 mg (RR = 1.35; 95% CI: [1.22, 1.49], *P* < 0.00001). Pooled results were heterogenous in 200 mg vs. placebo (*I*^2^ = 65%, *P*=0.03) and homogenous in 100 mg vs. placebo and 100 mg vs. 200 mg (*I*^2^ = 30%, *P*=0.23) (*I*^2^ = 45%, *P*=0.14), respectively. The heterogeneity was best resolved by excluding Pfizer (JADE compare trail) 2021.

### 4.4. EASI-90 Responders

Pooled analysis revealed that 100 and 200 mg abrocitinib significantly increased EASI-90 responders more than placebo (RR = 3.78; 95% CI: [2.53, 5.65], *P* < 0.00001) (RR = 5.72; 95% CI: [3.86, 8.49], *P* < 0.00001), respectively. Also, 200 mg abrocitinib was associated with higher EASI-90 responders more than 100 mg (RR = 1.51; 95% CI: [1.29, 1.78], *P* < 0.00001). Pooled results were homogenous (*I*^2^ = 0%, *P*=0.75) (*I*^2^ = 0%, *P*=0.57) (*I*^2^ = 45%, *P*=0.14), respectively.

**Figure 4:**
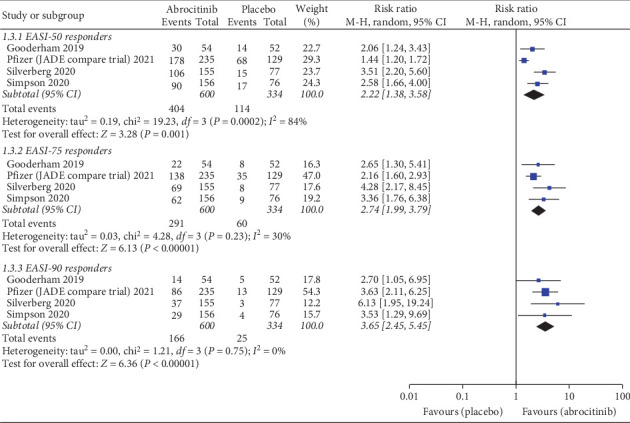
Forest plot of EASI-50, −75, and 90% responders (100 mg abrocitinib vs. placebo).

EASI-50 responders, EASI-75 responders, and EASI-90 responders for 100 mg abrocitinib vs. placebo are shown in Figure 4 while those for 200 mg abrocitinib vs. placebo and 100 mg vs. 200 mg abrocitinib are shown in Supplementary Figures 3 and 4.

### 4.5. Participants with at Least 4-Point Improvement in NRS

Pooled analysis revealed that 100 and 200 mg abrocitinib significantly increased the number of participants with at least 4-point improvements in NRS more than placebo (RR = 2.17; 95% CI: [1.51, 3.13], *P* < 0.0001) (RR = 2.60; 95% CI: [1.34, 5.04], *P*=0.005), respectively (Supplementary Figures 5 and 6), while there was no significant difference between 100 mg and 200 mg abrocitinib (RR = 0.87; 95% CI: [0.63, 1.20], *P*=0.39) (Supplementary Figure 7). Pooled results were heterogenous (*I*^2^ = 53%, *P*=0.09) (*I*^2^ = 86%, *P* < 0.0001) (*I*^2^ = 84%, *P*=0.0003), respectively, and the heterogeneity was best resolved by excluding Pfizer (JADE compare trail) 2021 in all three comparisons without effect on the significance except in 100 mg vs. 200 mg which became favoring 200 mg in terms of increasing the number of patients with at least 4-point improvement in NRS (RR = 0.74; 95% CI: [0.64, 0.86], *P*=0.001).

### 4.6. SCORAD Index

Pooled analysis revealed that 100 and 200 mg abrocitinib significantly reduced SCORAD index more than placebo (MD = (−13.33; 95% CI: [−14.62, −12.05], *P* < 0.00001) (MD = −24.70; 95% CI: [−25.98, −23.42], *P* < 0.00001), respectively. Also, 200 mg abrocitinib was associated with lower SCORAD index more than 100 mg (MD = −10.83; 95% CI: [−13.32, −8.34], *P* < 0.00001). Pooled results were homogenous (*I*^2^ = 0%, *P*=0.98) (*I*^2^ = 0%, *P*=0.32) (*I*^2^ = 35%, *P*=0.0.21), respectively.

### 4.7. % BSA

Pooled analysis revealed that 100 and 200 mg abrocitinib significantly reduced % BSA more than placebo (MD = −10.92; 95% CI: [−15.29, −6.55], *P* < 0.00001) (MD = −19.21; 95% CI: [−23.56, −14.87], *P* < 0.00001), respectively. Also, 200 mg abrocitinib was associated with lower % BSA more than 100 mg (MD = −8.33; 95% CI: [−12.06, −4.60], *P* < 0.0001). Pooled results were homogenous (*I*^2^ = 60%, *P*=0.12) (*I*^2^ = 59%, *P*=0.12) (*I*^2^ = 0%, *P*=0.98), respectively.

### 4.8. PSAAD Index

Pooled analysis revealed that 100 and 200 mg abrocitinib significantly reduced PSAAD more than placebo (MD = −1.23; 95% CI: [−1.54, −0.92], *P* < 0.00001) (MD = −2.08; 95% CI: [−2.39, −1.77], *P* < 0.00001), respectively. Also, 200 mg abrocitinib was associated with lower PSAAD more than 100 mg (MD = −0.83; 95% CI: [−1.09, −0.58], *P* < 0.00001). Pooled results were homogenous (*I*^2^ = 0%, *P*=0. 38) (*I*^2^ = 0%, *P*=0. 86) (*I*^2^ = 0%, *P*=0. 48), respectively.

### 4.9. POEM Index

Pooled analysis revealed that 100 and 200 mg abrocitinib significantly reduced % BSA more than placebo (MD = −6.72; 95% CI: [−7.79, −5.65], *P* < 0.00001) (MD = −7.33; 95% CI: [−8.39, −6.26], *P* < 0.00001), respectively, while there was no significant difference between 100 mg and 200 mg abrocitinib (MD = −0.73; 95% CI: [−2.19, 0.73], *P*=0.33). Pooled results were homogenous (*I*^2^ = 40%, *P*=0.19) (*I*^2^ = 0%, *P*=0.9) (*I*^2^ = 62%, *P*=0.07), respectively.

**Figure 5:**
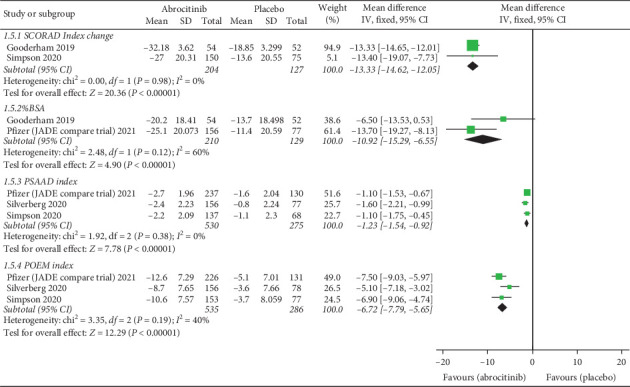
Forest plot of SCORAD index, % BSA, PSAAD index, and POEM index (100 mg abrocitinib vs. placebo).

SCORAD index, % BSA, PSAAD index, and POEM index for 100 mg abrocitinib vs. placebo are shown in Figure 5 while those for 200 mg abrocitinib vs. placebo and 100 mg vs. 200 mg abrocitinib are shown in (Supplementary Figures 8 and 9).

### 4.10. DLQI

Pooled analysis revealed that 100 and 200 mg abrocitinib significantly reduced PSAAD more than placebo (MD = −2.99; 95% CI: [−3.88, −2.09], *P* < 0.00001) (MD = -5.07; 95% CI: [−5.94, −4.20], *P* < 0.00001), respectively. Also, 200 mg abrocitinib was associated with lower PSAAD more than 100 mg (MD = −2.06; 95% CI [−2.81, −1.30], *P* < 0.00001). Pooled results were homogenous (*I*^2^ = 32%, *P*=0.23) (*I*^2^ = 0%, *P*=0.6) (*I*^2^ = 0%, *P*=0.67), respectively.

### 4.11. CDLQI

Pooled analysis revealed that 100 and 200 mg abrocitinib significantly reduced % BSA more than placebo (MD = −2.49; 95% CI: [−4.90, −0.07], *P*=0.04) (MD = −3.71; 95% CI: [−6.13, −1.30], *P*=0.003), respectively, while there was no significant difference between 100 mg and 200 mg abrocitinib (MD = −1.23; 95% CI: [−3.16, 0.71], *P*=0.21). Pooled results were homogenous (*I*^2^ = 0%, *P*=0.95) (*I*^2^ = 0%, *P*=0.62) (*I*^2^ = 0%, *P*=0.49), respectively.

DLQI and CDLQI for 100 mg abrocitinib vs. placebo, 200 mg abrocitinib vs. placebo, and 100 mg vs. 200 mg abrocitinib are shown in Supplementary Figures 10–12, respectively.

### 4.12. Serious Adverse Events of Any Cause

Pooled analysis revealed no difference between 100 or 200 mg abrocitinib and placebo (RR = 0.81; 95% CI: [0.38, 1.73], *P*=0.59) (RR = 0.50; 95% CI: [0.22, 1.16], *P*=0.11), respectively. Also, there was no significant difference between 100 mg and 200 mg abrocitinib (RR = 1.59; 95% CI: [0.72, 3.53], *P*=0.26). Pooled results were homogenous (*I*^2^ = 0%, *P*=0.87) (*I*^2^ = 0%, *P*=0.51) (*I*^2^ = 0%, *P*=0.74), respectively.

### 4.13. Nausea

Pooled analysis revealed that 100 or 200 mg abrocitinib was associated with higher incidence of nausea than placebo (RR = 2.83; 95% CI: [1.26, 6.35], *P*=0.01) (RR = 6.98; 95% CI: [3.27, 14.92], *P* < 0.00001), respectively. Moreover, 200 mg was associated with higher incidence of nausea than 100 mg (RR = 0.42; 95% CI: [0.29, 0.61], *P* < 0.00001). Pooled results were homogenous (*I*^2^ = 0%, *P*=0.89) (*I*^2^ = 0%, *P*=0.99) (*I*^2^ = 0%, *P*=0.57), respectively.

### 4.14. Headache

Pooled analysis revealed no difference between 100 abrocitinib and placebo (RR = 1.72; 95% CI: [0.91, 3.27], *P*=0.1), while 200 mg was associated with higher incidence of headache than placebo (RR = 2.22; 95% CI: [1.18, 4.16], *P*=0.01). Also, there was no significant difference between 100 mg and 200 mg abrocitinib (RR = 0.76; 95% CI: [0.50, 1.16], *P*=0.20). Pooled results were homogenous (*I*^2^ = 0%, *P*=0.49) (*I*^2^ = 0%, *P*=0.68) (*I*^2^ = 0%, *P*=0.85

), respectively.

### 4.15. Dermatitis Atopic

Pooled analysis revealed no difference between 100 abrocitinib and placebo (RR = 0.71; 95% CI: [0.47, 1.07], *P*=0.1), while 200 mg was associated with lower incidence of dermatitis atopic than placebo (RR = 0.50; 95% CI: [0.30, 0.82], *P*=0.007). Also, there was no significant difference between 100 mg and 200 mg abrocitinib (RR = 1.47; 95% CI: [0.94, 2.29], *P*=0.09). Pooled results were homogenous (*I*^2^ = 11%, *P*=0.34) (*I*^2^ = 10%, *P*=0.34) (*I*^2^ = 50%, *P*=0.11), respectively.

### 4.16. Nasopharyngitis

Pooled analysis revealed no difference between 100 or 200 mg abrocitinib and placebo (RR = 1.52; 95% CI: [0.96, 2.41], *P*=0.08) (RR = 1.08; 95% CI: [0.66, 1.76], *P*=0.75), respectively. Also, there was no significant difference between 100 mg and 200 mg abrocitinib (RR = 1.40; 95% CI: [0.98, 2.01], *P*=0.01). Pooled results were homogenous (*I*^2^ = 0%, *P*=0.81) (*I*^2^ = 0%, *P*=0.94) (*I*^2^ = 0%, *P*=0.85), respectively.

### 4.17. Upper Respiratory Tract Infection

Pooled analysis revealed no difference between 100 or 200 mg abrocitinib and placebo (RR = 1.20; 95% CI: [0.69, 2.06], *P*=0.52) (RR = 0.96; 95% CI: [0.55, 1.69], *P*=0.89), respectively. Also, there was no significant difference between 100 mg and 200 mg abrocitinib (RR = 1.30; 95% CI: [0.82, 2.06], *P*=0.27). Pooled results were homogenous (*I*^2^ = 0%, *P*=0.55) (*I*^2^ = 0%, *P*=0.99) (*I*^2^ = 23%, *P*=0.28), respectively.

**Figure 6:**
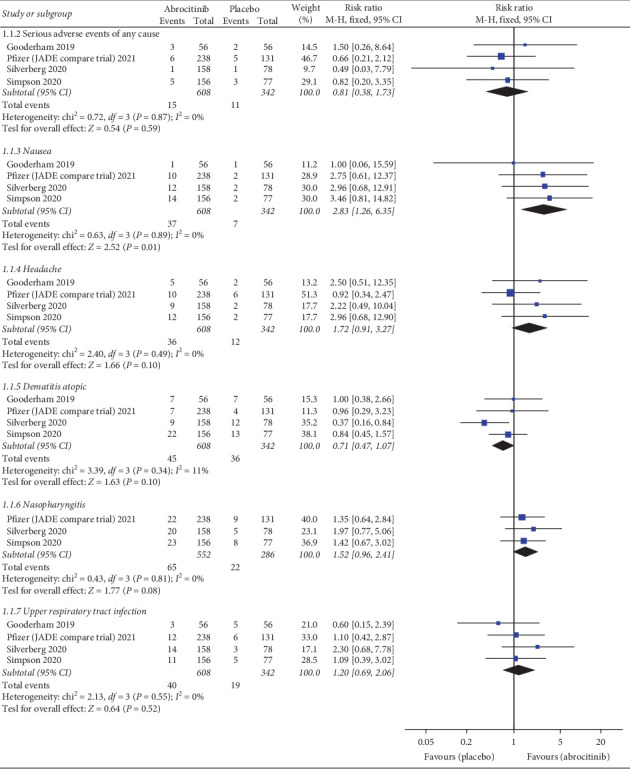
Forest plot of adverse events, serious adverse events of any cause, nausea, headache, dermatitis atopic, nasopharyngitis, and upper respiratory tract infection (100 mg abrocitinib vs. placebo).

Serious adverse events of any cause, nausea, headache, dermatitis, atopic, nasopharyngitis, and upper respiratory tract infection for 100 mg abrocitinib vs. placebo are shown in Figure 6 while those for 200 mg abrocitinib vs. placebo and 100 mg vs. 200 mg abrocitinib are shown in Supplementary Figures 13 and 14.

### 4.18. Death

Pooled analysis revealed no difference between 100 or 200 mg abrocitinib and placebo (RR = 0.858; 95% CI: [0.136, 5.412], *P*=0.87) (RR = 0.624; 95% CI: [0.088, 4.403], *P*=0.636), respectively (Supplementary Figures 15 and 16). Also, there was no significant difference between 100 mg and 200 mg abrocitinib (RR = 1.407; 95% CI: [0.223, 8.92], *P*=0.716) (Supplementary Figure 17). Pooled results were homogenous (*I*^2^ = 0%, *P*=0.97) (*I*^2^ = 0%, *P*=0.99) (*I*^2^ = 0%, *P*=0.96), respectively.

## 5. Discussion

We found that 100 mg and 200 mg abrocitinib were not associated with adverse events such as upper respiratory tract infection, nasopharyngitis, dermatitis atopic, any serious adverse events, and death. However, some specific adverse events may occur with 100 mg or 200 mg as nausea and headache. The prevalence of nausea and headache was higher in 200 mg than 100 mg. Regarding the efficacy, 100 mg and 200 mg were associated with higher IGA response, EASI-50% responders, EASI-75% responders, EASI-90% responders, number of participants with at least 4-point improvements in NRS, and quality of life measured by DLQI and CDLQI than placebo. Moreover, 100 mg and 200 mg were associated with lower SCORAD index, %BSA, PSAAD index, and POEM index than placebo. Abrocitinib 200 mg significantly increased IGA response, DLQI, EASI-50% responders, EASI-75% responders, EASI-90% responders than 100 mg while no difference regarding the number of participants with at least 4 points improvements in NRS, POEM index and CDLQI. Also, 200 mg abrocitinib significantly decreased SCORAD index, %BSA, PSAAD index than 100 mg.

JAK inhibitors are classed into several classes, as they can be used as immunomodulators as in case of DMARDS, tyrosine kinase inhibitors, and also inhibit cytokine activity modifying the immune system [21]. Tofacitinib is a treatment option for other autoimmune diseases and can also reduce pulmonary eosinophilia [21, 22]. It was recently reported that patients with moderate-to-severe active ulcerative colitis treated with tofacitinib were more likely to have an improved clinical response than placebo [23]. Baricitinib has also proved efficacy in patients with active rheumatoid arthritis (RA) [21]. Upadacitinib is indicated for treating moderate-to-severe active RA in adults who have responded inadequately to or were intolerant to one or more DMARDs [24].

Regarding the outcome of Investigator's Global Assessment (IGA) scale response, it is a 5-point modified assessment tool which evaluates whether treatment, clinicians, and regulators meet the need for a valid, clinically meaningful measure or not [25]. It can be used for evaluating plaque psoriasis or atopic dermatitis severity in clinical trials. Langley et al.[25] concluded that the 5-point IGA scale is a valid measure of disease severity. We depend on IGA in our analysis, and it showed a higher efficacy favoring abrocitinib over placebo. All included studies [13, 18, 20, 26] in the analysis observed a significant improvement in the IGA scale in the abrocitinib group. However, a higher dose (200 mg) showed a significant improvement rather than 100 mg.

Regarding the Eczema Area and Severity Index (EASI) score, it is an extensively validated scoring system that grades the physical signs of AD [27]. It is the core outcome for measuring the clinical signs of eczema in all trials. Leshem et al. [28] provided the first guide for interpreting the EASI score. ESAI enables translation of the numerical output into an AD global severity state that should be more meaningful to providers and patients. EASI has demonstrated adequate feasibility, further supporting its use in clinical trials [28]. ESAI was reported by all included trials favoring abrocitinib over placebo. However, a higher dose (200 mg) showed a significant improvement rather than 100 mg.

Regarding Pruritus Numerical Rating Scale (NRS) score, it is comprised of one item and represents the numbers 0 (“no itch”) to 10 (“worst imaginable itch”) [29]. Subjects are asked to rate the intensity of their itch using this scale with a simple format. It can be interpreted as follows: NRS = 0 indicates no pruritus, NRS<3 mild pruritus, NRS >3 < 7 moderate pruritus, NRS ≥7 < 9 severe pruritus, and NRS ≥9 very severe pruritus [29, 30]. The NRS is a similar tool and has also been validated to measure pain [30]. NRS showed a significant result in our analysis favoring abrocitinib over placebo. There are other different scales for assessment we used in the analysis as Scoring Atopic Dermatitis (SCORAD) and Patient-Oriented Eczema Measure (POEM) [31]. SCORAD is a clinical tool used to assess the extent and severity of eczema [31]. Schram et al. [31] detected that SCORAD had fair responsiveness to atopic eczema. POEM is a validated, patient-derived assessment measure for monitoring atopic eczema severity [32].

It is the term of the quality of life (QoL) score outcome, and it was initially created by American psychologist John Flanagan in the 1970s. The QOL score was originally a 15-item that measured five conceptual domains of quality of life which are as follows: material and physical well-being, relationships with other people, social, community and civic activities, personal development and fulfilment, and recreation [33]. The QOLS is a valid instrument for measuring the quality of life across patient groups and cultures [33]. The quality of the included clinical trials meets a trusted level of evidence. We analyzed all available outcomes reported in the included trials with a considerable number of patients. All detected heterogeneity could be resolved. However, there are some limitations; we could not assess the publication bias due to the limited number of the included studies. The measured outcomes were assessed after limited duration of follow-up with no availability of long-term follow-up periods. We recommend future clinical trials with more sample sizes and strictly follow-up for longer durations.

## 6. Conclusion

Finally, we concluded that abrocitinib in dose 100 mg or 200 mg is an effective, well tolerated, and promising drug in treating patients with moderate-to-severe atopic dermatitis. However, the analysis favored the efficacy of abrocitinib 200 mg over 100 mg, but side effects such as nausea and headache are likely to occur more with 200 mg.

## Figures and Tables

**Table 1:** Summary of the included RCTs.

Study ID	Study time and sites	Design and phase	Protocol NCT number	Total number of patients	Inclusion criteria	Study arms and number of patients	Dose, route, and regimen of abrocitinib	Duration of treatment	Atopic dermatitis severity	Study conclusion
Gooderham 2019	From April 15, 2016, to April 4, 2017, at 58 centers in Australia, Canada, Germany, Hungary, and the United States	RCT, phase 2	02780167	267	“Eligible patients were men or women aged 18 to 75 years with a clinical diagnosis of moderate to severe AD (percentage of affected body surface area (%BSA) ≥10; Investigator's global assessment (IGA) score ≥3; and eczema area and severity index (EASI) score ≥12) for 1 year or more before day 1 of the study and inadequate response to topical medications (topical corticosteroids or topical calcineurin inhibitors) for 4 weeks or more (based on investigator's judgment) or inability to receive topical treatment within 12 months before the first dose of study drug because it was medically inadvisable (e.g., application to a large %BSA, which is associated with increased risk for systemic absorption and suppression of the hypothalamic-pituitary-adrenal axis, and cutaneous adverse effects such as burning or stinging sensations with topical calcineurin inhibitors or skin atrophy, purpura, telangiectasia, and striae with chronic use of topical corticosteroids)”	Abrocitinib 10 mg, 49 Abrocitinib 30 mg, 51 Abrocitinib 100 mg, 56 Abrocitinib 200 mg, 55 Placebo, 56	(10, 30, 100, 200) mg, oral, once daily	12 weeks	Patients with moderate-to-severe atopic dermatitis	“Once-daily oral abrocitinib was effective and well tolerated for short-term use in adults with moderate to severe atopic dermatitis. Additional trials are necessary to evaluate long-term efficacy and safety”

Silverberg 2020	From June 29, 2018, to August 13, 2019, in Australia, Bulgaria, Canada, China, Czechia, Germany, Hungary, Japan, south, korea, Latvia, Poland, United Kingdom, and the United States.	RCT, phase 3	03575871	391	“Eligible patients were 12 years or older, with body weight of at least 40 kg. Adolescent patients younger than 18 years (or country-specific age of majority) were eligible on a country-by-country basis as approved by the country or regulatory/health authority. Eligible patients had a confirmed diagnosis of chronic AD23 for at least 1 year before the first dose of study drug and moderate-to-severe AD (Investigator's global assessment (IGA) score ≥3, eczema area and severity index (EASI) score 24 ≥ 16, affected body surface area ≥10%, and peak pruritus numerical rating scale (PP-NRS, used with permission of regeneron pharmaceuticals, inc, and sanofi SA) score 25 ≥ 4) at the baseline visit. Eligible patients also had a documented recent history (within 6 months before screening) of inadequate response to treatment with topical corticosteroids or topical calcineurin inhibitors given for at least 4 weeks, a history of topical AD treatments being considered medically inadvisable, or a history of receiving systemic therapies for AD”	Abrocitinib 100 mg, 158 Abrocitinib 200 mg, 155 Placebo, 78	(100, 200) mg, oral, once daily	12 weeks	Patients with moderate-to-severe atopic dermatitis	“Monotherapy with once daily oral abrocitinib was effective and well tolerated in adolescents and adults with moderate-to-severe AD”

Simpson 2020	From Dec 7, 2017, to March 26, 2019, in 69 hospitals and clinics in Australia, Canada, europe, and the United States.	RCT, phase 3	03349060	387	“All eligible patients had a confirmed diagnosis of atopic dermatitis for at least 1 year before randomization (according to hanifin and rajka diagnostic criteria 21); had moderate -to- severe atopic dermatitis (investigator global assessment score ≥3, EASI score ≥16, percentage of body surface area affected ≥10%, and peak pruritus numerical rating scale [PP-NRS] score ≥4) at the baseline visit. The PP-NRS score was used with the permission of regeneron pharmaceuticals (tarrytown, NY, USA) and sanofi SA (paris, France).22 eligible patients also had a documented recent history (in the 6 months before screening) of inadequate response to treatment with topical corticosteroids or topical calcineurin inhibitors given for at least 4 weeks, or were patients for whom topical treatments were otherwise medically inadvisable, or required systemic therapies to control their disease”	Abrocitinib 100 mg, 156 Abrocitinib 200 mg, 154 Placebo, 77	(100, 200) mg, oral, once daily	12 weeks	Patients with moderate-to-severe atopic dermatitis	“Monotherapy with oral abrocitinib once daily was effective and well tolerated in adolescents and adults with moderate-to-severe atopic dermatitis”

Pfizer (JADE compare trial) 2021		RCT, phase 3	03720470	837	“Male or female subjects aged 18 years or older at the time of informed consent. Diagnosis of atopic dermatitis (AD) for at least 1 year and current status of moderate to severe disease (>= the following scores: BSA 10%, IGA 3, EASI 16, pruritus NRS severity 4). Documented recent history (within 6 months before the screening visit) of inadequate response to treatment with medicated topical therapy for AD for at least 4 weeks, or who have required systemic therapies for control of their disease. Must be willing and able to comply with standardized background topical therapy, as per protocol guidelines throughout the study female subjects who are of childbearing potential must not be intending to become pregnant, currently pregnant, or lactating. The following conditions apply: - Female subjects of childbearing potential must have a confirmed negative pregnancy test prior to randomization; - female subjects of childbearing potential must agree to use a highly effective method of contraception for the duration of the active treatment period and for at least 28 days after the last dose of investigational product. Female subjects of non-childbearing potential must meet at least 1 of the following criteria: have undergone a documented hysterectomy and/or bilateral oophorectomy; have medically confirmed ovarian failure; or achieved postmenopausal status, defined as follows: Cessation of regular menses for at least 12 consecutive months with no alternative pathological or physiological cause and have a serum follicle stimulating hormone (FSH) level confirming the postmenopausal state. All other female subjects (including female subjects with tubal ligations) are considered to be of childbearing potential. If receiving concomitant medications for any reason other than AD, must be on a stable regimen prior to day 1 and through the duration of the study”	First 16 weeks final 4 weeks (16-20)	Abrocitinib 100 mg, 238 Abrocitinib 200 mg, 226 Placebo, 131 Dupilumab 300 mg, 242 -abrocitinib 100 mg, 238 (after 16 weeks abrocitinib 100 mg) -Abrocitinib 200 mg, 226 (after 16 weeks abrocitinib 100 mg) -Abrocitinib 100 mg, 60 (after 16 weeks placebo) -Abrocitinib 200 mg, 57 (after 16 weeks placebo) -Placebo, 242 (after 16 weeks dupilumab)	(100, 200) mg, oral, once daily	20 weeks (initial 16 then 4)	Patients with moderate-to-severe atopic dermatitis

**Table 2:** Baseline characteristics of the enrolled patients in the included RCTs.

Study ID		Study groups	Number of patients	Age, mean (SD)	Sex (male), no. (%)	Race, no. (%)					Disease duration, mean (SD), y	Investigator's global assessment (IGA) grade, no. (%)		Eczema area and severity index (EASI) score, mean (SD)	% body surface area (BSA) affected, mean (SD)	Pruritus numeric rating scale (NRS) score, mean (SD)	Scoring atopic dermatitis (SCORAD), mean (SD)	Pruritus and symptoms assessment for atopic dermatitis (PSAAD), mean (SD)	Patient-oriented eczema measure (POEM), mean (SD)	Dermatology life quality index (DLQI), mean (SD), patients	Children's dermatology life quality index (CDLQI), mean (SD), patients	Previous medications for AD
						White	Black	Asian	Others	Not reported		Moderate (grade 3)	Severe (grade 4)									
Gooderham 2019		Abrocitinib 10 mg Abrocitinib 30 mg Abrocitinib 100 mg Abrocitinib 200 mg Placebo	49 51 56 55 56	44.3 (15.9) 37.6 (15.9) 41.1 (15.6) 38.7 (17.6) 42.6 (15.1)	21 (42.9) 22 (43.1) 31 (55.4) 28 (50.9) 21 (37.5)	38 (77.6) 39 (76.5) 40 (71.4) 37 (67.3) 40 (71.4)	5 (10.2) 4 (7.8) 7 (12.5) 13 (23.6) 10 (17.9)	5 (10.2 5 (9.8) ) 8 (14.3) 5 (9.1) 4 (7.1)	1 (2.0) 3 (5.9) 1 (1.8) 0 (0) 2 (3.6)	—	30.3 (14.7) 20.5 (16.35) 23.8 (16.4) 19.6 (16.73) 25.6 (16.5)	27 (55.1) 28 (56.0) 29 (52.7) 34 (63.0) 34 (61.8)	22 (44.9) 22 (44.0) 26 (47.3 20 (37.0)) 21 (38.2)	28.1 (13.1) 22.1 (10.7) 26.7 (11.8) 24.6 (13.5) 25.4 (12.9)	44.2 (22.7) 34.1 (20.8) 41.9 (22.3) 38.0 (23.3) 40.1 (22.3)	7.6 (1.7) 7.6 (1.9) 7.4 (2.2) 6.9 (2.7) 7.6 (1.8)	65.3 (13.2) 62.4 (13.0) 65.4 (13.7) 62.7 (13.7) 65.0 (12.1)	—	—	—	—	Topical corticosteroids or topical calcineurin inhibitors

Silverberg 2020		Abrocitinib 100 mg Abrocitinib 200 mg Placebo	158 155 78	37.4 (15.8) 33.5 (14.7) 33.4 (13.8)	94 (59.5) 88 (56.8) 47 (60.3)	101(63.9) 91 (58.7) 40 (51.3)	9 (5.7) 6 (3.9) 6 (7.7)	46 (29.1) 54 (34.8) 29 (37.2)	1 (0.6) 2 (1.3) 1 (1.3)	1 (0.6) 2 (1.3) 2 (2.6)	21.1 (14.8) 20.5 (14.8) 21.7 (14.3)	107 (67.7) 106 (68.4) 52 (66.7)	51 (32.3) 49 (31.6) 26 (33.3)	28.4 (11.2) 29.0 (12.4) 28.0 (10.2)	48.7 (21.4) 47.7 (22.3) 48.2 (20.8)	7.1 (1.6) 7.0 (1.6) 6.7 (1.9)	63.8 (11.4) 64.1 (13.1) 64.3 (12.4)	5.4 (2.1) 5.2 (2.0) 5.1 (2.1)	20.9 (5.7) 19.7 (5.7) 19.2 (5.5)	15.4 (7.3), 140 14.8 (6.0), 139 15.0 (7.1), 70	13.8 (5.8), 16 12.9 (5.7), 15 10.1 (3.8), 8	Anti-inflammatory topical agents alone (226), systemic agents and/or topical agents (162), dupilumab (14)

Simpson 2020		Abrocitinib 100 mg Abrocitinib 200 mg Placebo	156 154 77	32·6 (15·4) 33·0 (17·4) 31·5 (14·4)	90 (58) 81 (53) 49 (64)	113 (72) 104 (68) 62 (81)	15 (10) 11 (7) 6 (8)	26 (17) 26 (17) 6 (8)	2 (1) 11 (7) 2 (3)	0 (0) 2 (1%) 1 (1%)	24·9 (16·1) 22·7 (14·5) 22·5 (14·4)	92 (59) 91 (59) 46 (60)	64 (41) 63 (41) 31 (40)	31·3 (13·6) 30·6 (14·1) 28·7 (12·5)	50·8 (23·4) 49·9 (24·4) 47·4 (22·7)	6·9 (2·0) 7·1 (1·9) 7·0 (1·8)	67·1 (13·7) 64·3 (13·1) 64·5 (13·2)	5·3 (2·3) 5·4 (2·1) 5·5 (2·0)	19·5 (6·5) 19·6 (5·9) 19·9 (6·1)	14·6 (6·5), 121 14·6 (6·8), 119 13·9 (7·3), 60	11·7 (6·6), 32 13·2 (5·5), 32 13·6 (7·0), 16	Anti-inflammatory topical agents alone (185), systemic agents and/or topical agents (187), dupilumab (30)

Pfizer (JADE compare trial) 2021	First 16 weeks	Abrocitinib 100 mg Abrocitinib 200 mg -Placebo Dupilumab 300 mg	238 226 131 242	−(224) 18–65 (14) >=65 −(211) 18–65 (15) >=65 −(121) 18–65 (10) >=65 −(227) 18–65 (15) >=65	120 (50.4) 104 (46) 77 (58.8) 108 (44.6)	182 (76.5) 161 (71.2) 87 (66.4) 176 (72.7)	6 (2.5) 9 (4) 6 (4.6) 14 (5.8)	48 (20.2) 53 (23.5) 31 (23.7) 46 (19.0)	2 (0.0084) 2 (0.0088) 4 (0.031) 4 (0.0165)	0 (0.0) 1 (0.4) 3 (2.3) 2 (0.8)	—	—	—	—	—	—	—	—	—	—	—	—
	Final 4 weeks (16-20)	-Abrocitinib 100 mg (after 16 weeks abrocitinib 100 mg) -Abrocitinib 200 mg (after 16 weeks placebo) Abrocitinib 100 mg (after 16 weeks placebo) Abrocitinib 200 mg (after 16 weeks placebo) Placebo (after 16 weeks dupilumab)	238 226 60 57 242	−(224) 18–65 (14) >=65 −(121) 18–65 (10) >=65 −(121) 18–65 (10) >=65 −(227) 18–65 (15) >=65	120(50.4) 104(46) 77 (58.8) 77 (58.8) 108 (44.6)	182 (76.5) 161 (71.2) 87 (66.4) 87 (66.4) 176(72.7)	6 (2.5) 9 (4) 6 (4.6) 6 (4.6) 14 (5.8)	48 (20.2) 53 (23.5) 31 (23.7) 31 (23.7) 46 (19.0)	2 (0.0084) 2 (0.0088) 4 (0.031) 4 (0.031) 4 (0.0165)	0 (0.0) 1 (0.4) 3 (2.3) 3 (2.3) 2 (0.8)	—	—	—	—	—	—	—	—	—	—	—	—

## Data Availability

All the data supporting the results of this study are available from the corresponding author upon request.

## Supplementary Materials

Supplementary Materials This file includes 17 figures in the form of forest plots of all measured outcomes with the legend of each figure, IGA response, EASI-50 responders, EASI-75 responders, EASI-90 responders, participants with at least 4-point improvement in NRS, SCORAD index, % BSA, PSAAD index, POEM index, DLQI, CDLQI, nausea, headache, dermatitis atopic, nasopharyngitis, and upper respiratory tract infection.
