# Corrigendum to “Effectiveness and Safety of Abrocitinib in Patients with Moderate-to-Severe Atopic Dermatitis: A Systematic Review and Meta-Analysis of Randomized Clinical Trials”

**DOI:** 10.1155/drp/9753406

**Published:** 2025-09-16

H. A. Fadlalmola, M. S. Albadrani, A. M. Elhusein, W. E. Mohamedsalih, D. S. V. Swamy, and D. M. Mamanao, “Effectiveness and Safety of Abrocitinib in Patients with Moderate-to-Severe Atopic Dermatitis: A Systematic Review and Meta-Analysis of Randomized Clinical Trials,” *Dermatology Research and Practice* 2021 (2021): 8382761, https://doi.org/10.1155/2021/8382761.

In the article titled “Effectiveness and Safety of Abrocitinib in Patients with Moderate-to-Severe Atopic Dermatitis: A Systematic Review and Meta-Analysis of Randomized Clinical Trials,” there was an error in affiliation number 3. In the corrected affiliation, the “*Bisha University*” should be changed to “*University of Bisha*” in the article. The corrected affiliation appears below:

“*College of Applied Medical Science, University of Bisha, Bisha, Saudi Arabia*”

We apologize for this error.

