# Supplementary material for: Effectiveness and Safety of Abrocitinib in Patients with Moderate-to-Severe Atopic Dermatitis: A Systematic Review and Meta-Analysis of Randomized Clinical Trials
Source: Dermatol Res Pract. 2021 Jun 23;2021:8382761. doi: 10.1155/2021/8382761 (PMC8245258; doi:10.1155/2021/8382761)
Supplement: Supplementary Materials — This file includes 17 figures in the form of forest plots of all measured outcomes with the legend of each figure, IGA response, EASI-50 responders, EASI-75 responders, EASI-90 responders, participants with at least 4-point improvement in NRS, SCORAD index, % BSA, PSAAD index, POEM index, DLQI, CDLQI, nausea, headache, dermatitis atopic, nasopharyngitis, and upper respiratory tract infection. [file 8382761.f1.docx]

| **Study ID** | **Risk of bias** | **Judgment of the authors** |
| --- | --- | --- |
| **(Gooderham 2019)** |  |  |
| **Random sequence generation (selection bias)** | Low risk | Randomization was via an interactive response technology system. Blinded abrocitinib and placebo  tablets were delivered to the study sites in blister packs. |
| **Allocation concealment (selection bias)** | Low risk | Randomization was via an interactive response technology system. Blinded abrocitinib and placebo  tablets were delivered to the study sites in blister packs. |
| **Blinding of participants and personnel (performance bias)** | Low risk | Patients, investigators, and sponsors were blinded to study treatment. |
| **Blinding of outcome assessment (Detection bias)** | Low risk | Patients, investigators, and sponsors were blinded to study treatment. |
| **Incomplete outcome data (attrition bias)** | Low risk | Safety was assessed in the safety analysis set, which included all randomized patients who received 1 dose or more of the study drug. Efficacy was assessed in the full analysis set, which was a modified intention-to-treat population that included all patients who received 1 dose or more of the study drug except for 4 patients from 1 site. |
| **Selective reporting (reporting bias)** | High risk | Not all of the study’s pre-specified primary outcomes have been reported; |
| **Other Bias** | High risk | Dr Gooderham reported receiving grants, personal fees, and/or nonfinancial support from AbbVie, Amgen, Akros, Arcutis, Bristol-Myers Squibb, Boehringer Ingelheim, Celgene, Coherus, Dermira, Eli Lilly, Galderma, Glenmark, Janssen Pharmaceuticals, Kyowa Kirin, LEO Pharma, MedImmune, Merck, Novartis, Pfizer, Roche, Sanofi Genzyme, Regeneron, UCB, and Valeant. Dr Bissonnette reported receiving grants, personal fees, and/or nonfinancial support from Aquinox Pharma, Antiobix, Asana, Astellas, Brickell Biotech,  Dermavant, Dermira, Dignity Sciences, Galderma, Glenmark, GSK-Stiefel, F. Hoffman-La Roche Ltd,  LEO Pharma, Neokera, Pfizer, Regeneron, and Vitae; and being a shareholder of lnnovaderm Research. Drs Beebe, Zhang, Banfield, Zhu, Papacharalambous, Vincent, and Peeva reported being employees of Pfizer Inc. |
|  |  |  |
| **(Silverberg 2020)** |  |  |
| **Random sequence generation (selection bias)** | Low risk | Patients were randomly assigned (2:2:1) to receive once-daily oral abrocitinib in 200-mg or 100-mg doses or matching placebo for 12 weeks with a computer-generated randomization schedule using interactive response technology (Almac Group). |
| **Allocation concealment (selection bias)** | Low risk | Patients were randomly assigned (2:2:1) to receive once-daily oral abrocitinib in 200-mg or 100-mg doses or matching placebo for 12 weeks with a computer-generated randomization schedule using interactive response technology (Almac Group). |
| **Blinding of participants and personnel (performance bias)** | Low risk | Patients, investigators, and sponsors were blinded to study treatment. |
| **Blinding of outcome assessment (Detection bias)** | Low risk | Patients, investigators, and sponsors were blinded to study treatment. |
| **Incomplete outcome data (attrition bias)** | Low risk | No missing outcome data as all patients were included in full analysis set |
| **Selective reporting (reporting bias)** | Low risk | The study protocol is available and all of the study’s pre-specified (primary and secondary) outcomes that are of interest in the review have been reported in the pre-specified way. |
| **Other Bias** | High risk | Dr Silverberg reported serving as an investigator for Celgene Corporation, Eli Lilly and Company, F. Hoffmann-LaRoche, Menlo Therapeutics, Realm Therapeutics PLC, Regeneron Pharmaceuticals, Inc, and Sanofi SA; as a consultant for Pfizer Inc and the study was funded and managed by Pfizer Inc. |
|  |  |  |
| **(Simpson 2020)** |  |  |
| **Random sequence generation (selection bias)** | Low risk | Patients were randomly assigned (2:2:1) to receive oral abrocitinib 100 mg, abrocitinib 200 mg, or matching placebo, using a central randomization scheme provided by an interactive response technology system. |
| **Allocation concealment (selection bias)** | Low risk | Patients were randomly assigned (2:2:1) to receive oral abrocitinib 100 mg, abrocitinib 200 mg, or matching placebo, using a central randomization scheme provided by an interactive response technology system. |
| **Blinding of participants and personnel (performance bias)** | Low risk | Patients, investigators, and the funder of the study were masked to study treatment. The placebo tablets were identical to the abrocitinib 100 mg tablets in size, color, shape, and odor. |
| **Blinding of outcome assessment (Detection bias)** | Low risk | Patients, investigators, and the funder of the study were masked to study treatment. The placebo tablets were identical to the abrocitinib 100 mg tablets in size, color, shape, and odor. |
| **Incomplete outcome data (attrition bias)** | Low risk | The primary analysis population for efficacy data was the full analysis set, which included all randomized  patients who received at least one dose of study medication. |
| **Selective reporting (reporting bias)** | Low risk | The study protocol is available and all of the study’s pre-specified (primary and secondary) outcomes that are of interest in the review have been reported in the pre-specified way. |
| **Other Bias** | High risk | ELS is a consultant for Pfizer; reports personal fees from AbbVie, Celgene, Dermira Pharmaceuticals, Galderma, Genentech, Menlo Therapeutics, LEO Pharma, Sanofi Genzyme, Valeant Pharmaceutical, Dermavant, and Pierre Fabre Dermo Cosmetique; grants and personal fees from Anacor Pharma, Eli Lilly, GlaxoSmithKline, Pfizer, Regeneron Pharmaceuticals, and Novartis; and grants from MedImmune, Tioga Pharmaceuticals, and Vanda Pharmaceuticals. RS has been principal investigator in clinical trials, served on advisory boards, received personal fees or non­financial support from Pfizer, AbbVie, Amgen, Bristol­Myers Squibb, Boehringer Ingelheim, Botanix, Celgene, Coherus, Dermira, Eli Lilly, Galderma, Janssen, LEO Pharma, Principia, MedImmune, Merck, Novartis, Roche, Sanofi­Genzyme, Regeneron, UCB, and Valeant. AW has been an advisor, speaker, or investigator for Pfizer during the conduct of the study; and reports  personal fees from AbbVie, Chugai, Galderma, Eli Lilly, MedImmune, Novartis, Pfizer, Regeneron, and Sanofi­Aventis; and grants and personal fees from LEO Pharma, outside the submitted work. RA is a consultant for Pfizer, LEO Pharma, Biofrontera, and Sanofi; and has received speaker fees from Alma Lasers, Biofrontera, Galderma, LEO Pharma, and Sanofi. MC reports grants from Sheffield Teaching Hospitals, during the conduct of the study; grants and personal fees from Sanofi­Genzyme/Regeneron, Pfizer, LEO Pharma, L’Oreal, La Roche­Possay, Johnson & Johnson, Perrigo/ACO Nordic, and Hyphens Pharma; and grants from Galapagos, outside the submitted work. TB reports personal fees from Pfizer, during the conduct of the study; personal fees from Lilly, AbbVie, Sanofi, LEO Pharma, and Galapagos; and grants from Glenmark and Galderma, outside the submitted work. JPT reports personal fees from Pfizer; and is an advisor, investigator, and speaker for Pfizer, AbbVie, Eli Lilly, LEO Pharma, and Sanofi­Genzyme. GY reports grants,  personal fees, and non­financial support from Pfizer during the conduct of the study; grants from LEO Pharma and Sun Pharmaceutical Industries; grants and personal fees from SanofiRegeneron, Menlo Therapeutics, and Kiniksa; personal fees and non­financial support from Galderma; and personal fees from Sienna Biopharmaceuticals, Trevi Therapeutics, Bellus, Bayer, AbbVie, CeraVe, Novartis, Eli Lilly, and Ortho, outside the submitted work. CFl reports grants from the EU Innovative Medicines Initiative BIOMAP  consortium, the UK National Institute for Health Research for TREAT trial, and the British Skin Foundation for UK­Irish Atopic Eczema Systemic Therapy Register, outside the submitted work. CM reports grants from Pfizer, during the conduct of the study; grants and personal fees from Lilly Pharma, Sanofi­Regeneron, and AbbVie; and grants from Asana Bioscience and Glenmark, outside the submitted work. |
|  |  |  |
| **(Pfizer,** **JADE**  **Compare trial 2021)** |  |  |
| **Random sequence generation (selection bias)** | Unclear | Insufficient information about the sequence generation process to permit judgement of ‘Yes’ or ‘No’ |
| **Allocation concealment (selection bias)** | Low risk | Subjects who are randomized to receive dupilumab injections every two weeks will also receive oral placebo to be taken once daily until Week 16 and will then continue to receive only the oral placebo for 4 weeks. Subjects who are randomized to the placebo arms, will receive both daily oral placebo and injectable placebo every two weeks until Week 16, after which they will receive either 100 mg or 200 mg of PF-04965842 taken orally once daily for 4 weeks, dependent upon which arm they have been allocated to. |
| **Blinding of participants and personnel (performance bias)** | Low risk | Subjects who are randomized to receive dupilumab injections every two weeks will also receive oral placebo to be taken once daily until Week 16 and will then continue to receive only the oral placebo for 4 weeks. Subjects who are randomized to the placebo arms, will receive both daily oral placebo and injectable placebo every two weeks until Week 16, after which they will receive either 100 mg or 200 mg of PF-04965842 taken orally once daily for 4 weeks, dependent upon which arm they have been allocated to. |
| **Blinding of outcome assessment (Detection bias)** | Low risk | Insufficient information to permit judgement of ‘Yes’ or ‘No’ |
| **Incomplete outcome data (attrition bias)** | Low risk | The primary analysis population for efficacy data was the full analysis set, which included all randomized  patients who received at least one dose of study medication. |
| **Selective reporting (reporting bias)** | Unclear | Not applicable |
| **Other Bias** | Unclear | Insufficient information to permit judgement of ‘Yes’ or ‘No’ |

**Table S1, Risk of bias assessment of the included trials according to Cochrane tool.**

**Supplementary figures ligands:**

**Supplementary figure S1:** Forest plot of IGA response (200mg abrocitinib vs placebo)

**Supplementary figure S2:** Forest plot of IGA response (100mg vs 200mg abrocitinib)

**Supplementary figure S3:** Forest plot of EASI 50, 75, 90% responders (200mg abrocitinib vs placebo)

**Supplementary figure S4:** Forest plot of EASI 50, 75, 90% responders (100mg vs 200mg abrocitinib)

**Supplementary figure S5:** Forest plot of number of participants with at least 4 points improvement in NRS (100mg abrocitinib vs placebo)

**Supplementary figure S6:** Forest plot of number of participants with at least 4 points improvement in NRS (200mg abrocitinib vs placebo)

**Supplementary figure S7:** Forest plot of number of participants with at least 4 points improvement in NRS (100mg vs 200mg abrocitinib)

**Supplementary figure S8:** Forest plot of SCORAD index, % BSA, PSAAD index, and POEM index (200mg abrocitinib vs placebo)

**Supplementary figure S9:** Forest plot of SCORAD index, % BSA, PSAAD index, and POEM index (100mg vs 200mg abrocitinib)

**Supplementary figure S10:** Forest plot of DLQI, CDLQI (100mg abrocitinib vs placebo)

**Supplementary figure S11:** Forest plot of DLQI, CDLQI (200mg abrocitinib vs placebo)

**Supplementary figure S12:** Forest plot of DLQI, CDLQI (100mg vs 200mg abrocitinib)

**Supplementary figure S13:** Forest plot of adverse events - serious adverse events of any cause, nausea, headache, dermatitis atopic, nasopharyngitis, and upper respiratory tract infection - (200mg abrocitinib vs placebo)

**Supplementary figure S14:** Forest plot of adverse events - serious adverse events of any cause, nausea, headache, dermatitis atopic, nasopharyngitis, and upper respiratory tract infection - (100mg vs 200mg abrocitinib)

**Supplementary figure S15:** Forest plot of death (100mg abrocitinib vs placebo)

**Supplementary figure S16:** Forest plot of death (200mg abrocitinib vs placebo)

**Supplementary figure S17:** Forest plot of death (100mg vs 200mg abrocitinib)

**Figure S1**

**
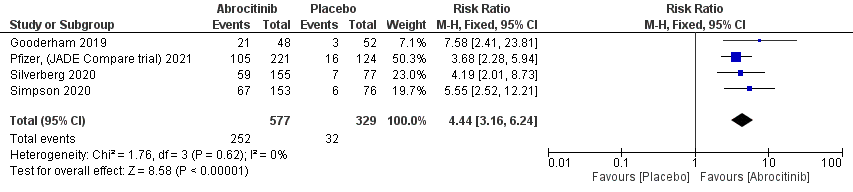
**

**Figure S2**

**
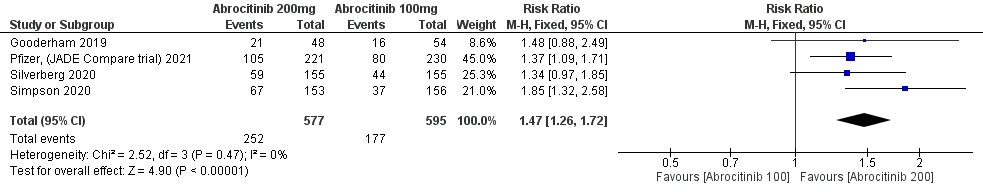
**

**Figure S3**

**
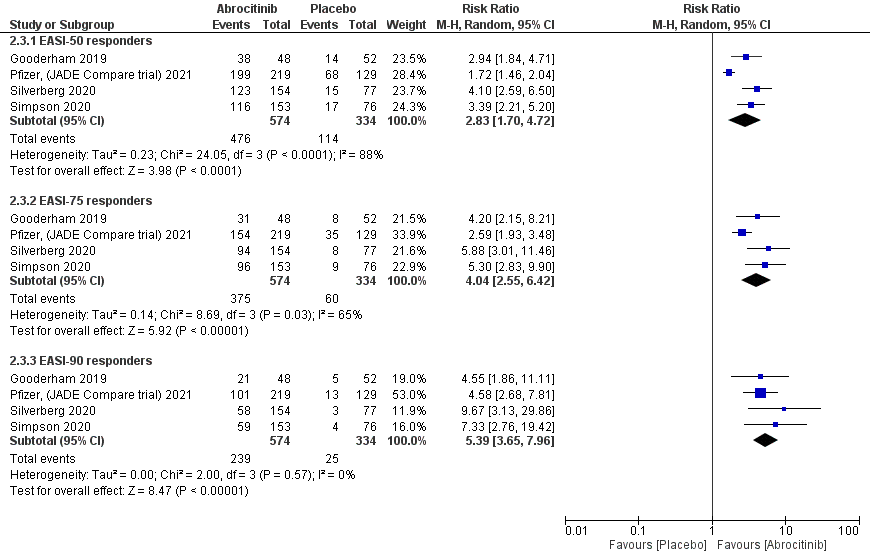
**

**Figure S4**

**
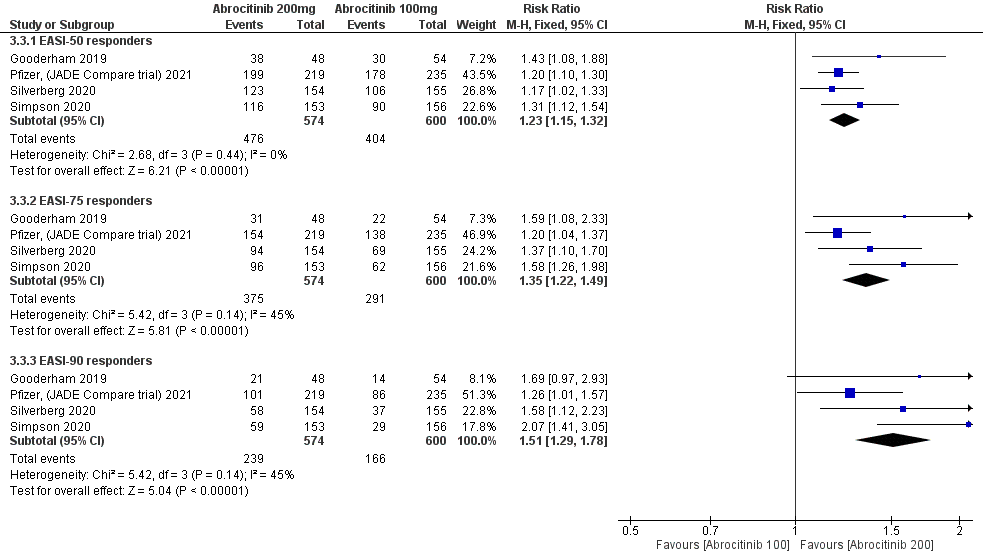
**

**Figure S5**

**
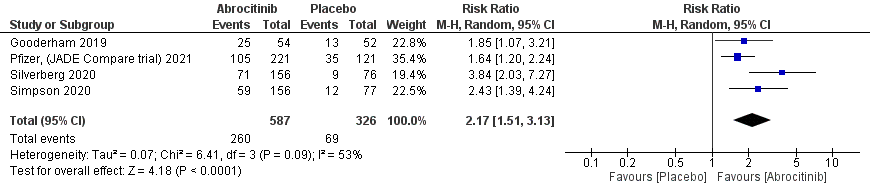
**

**Figure S6**

**
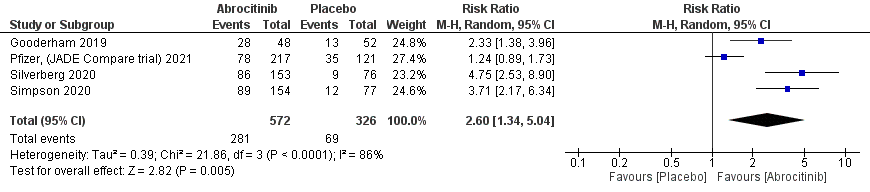
**

**Figure S7**

**
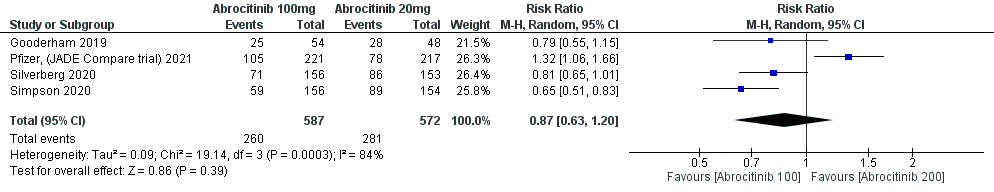
**

**Figure S8**

**
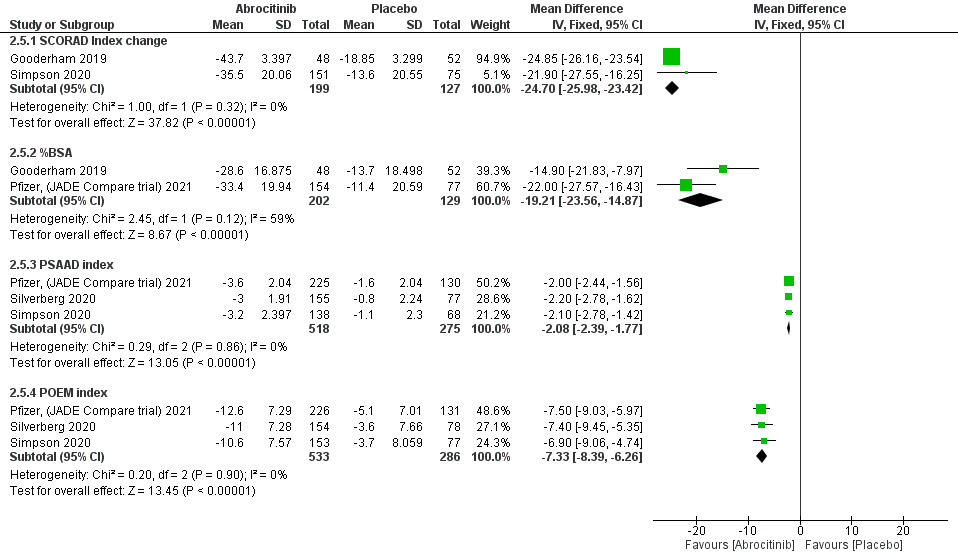
**

**Figure S9**

**
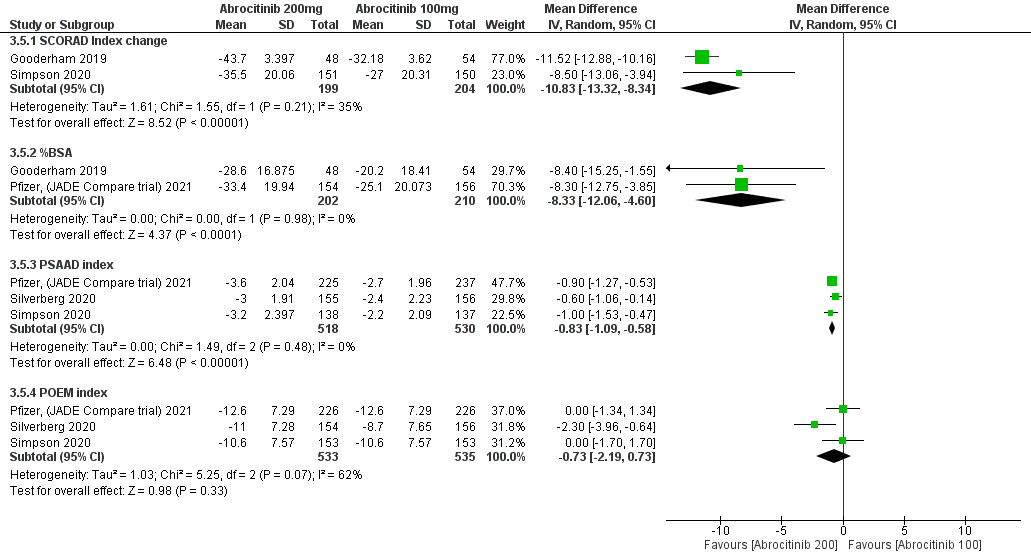
**

**Figure S10**

**
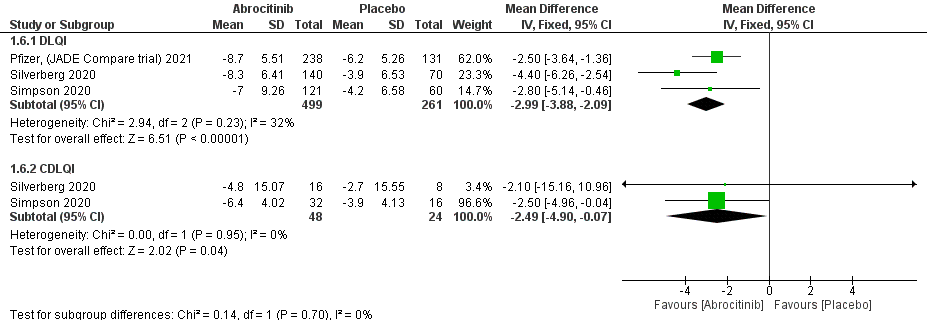
**

**Figure S11**

**
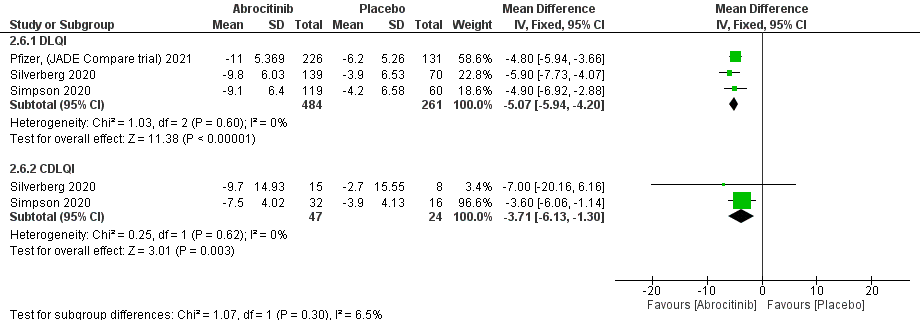
**

**Figure S12**

**
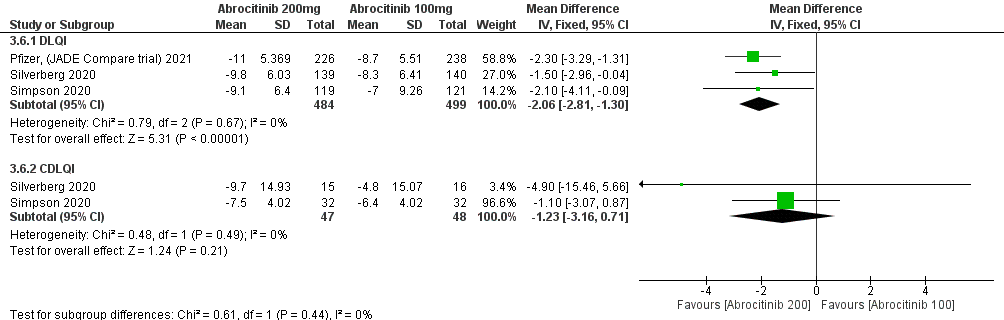
**

**Figure S13**

**
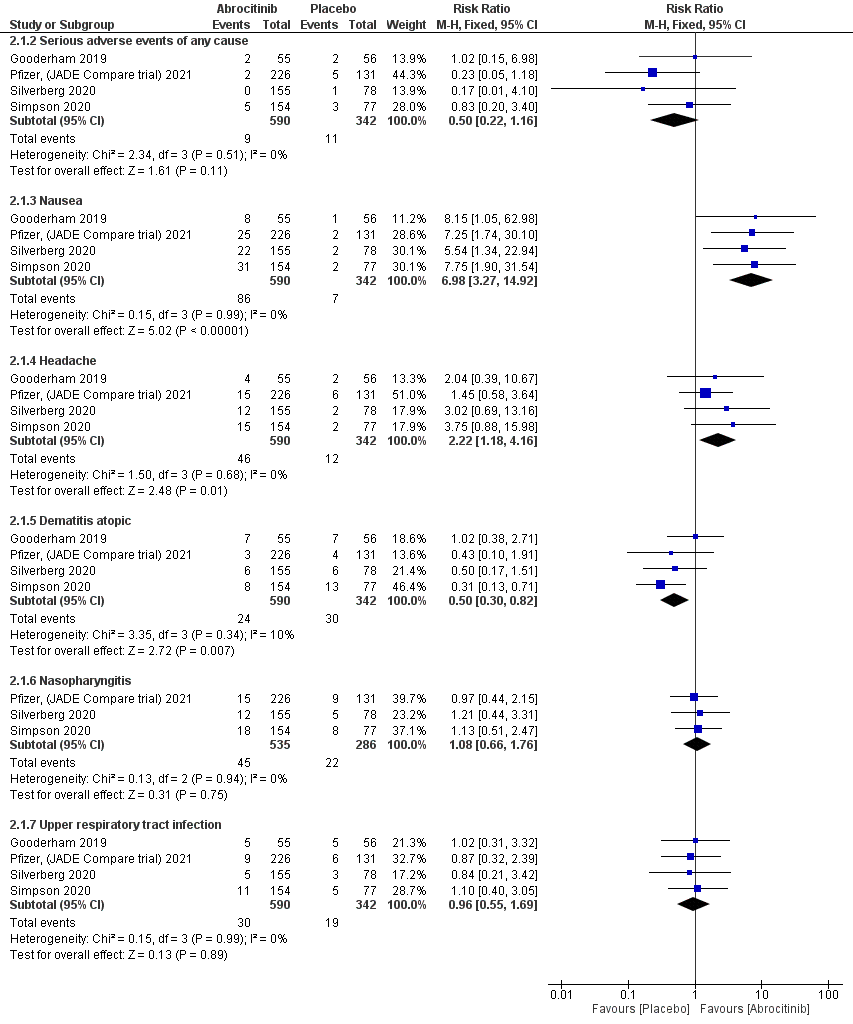
**

**Figure S14**

**
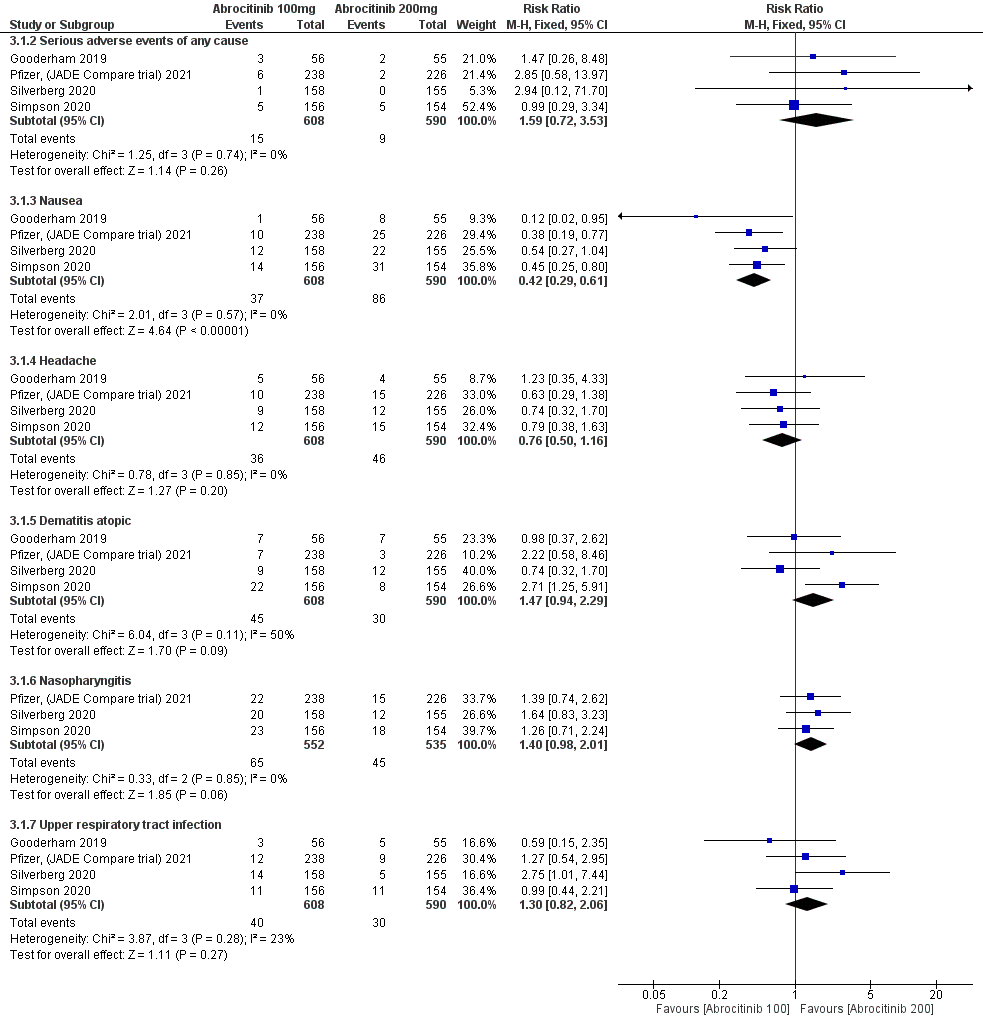
**

**Figure S15**

**
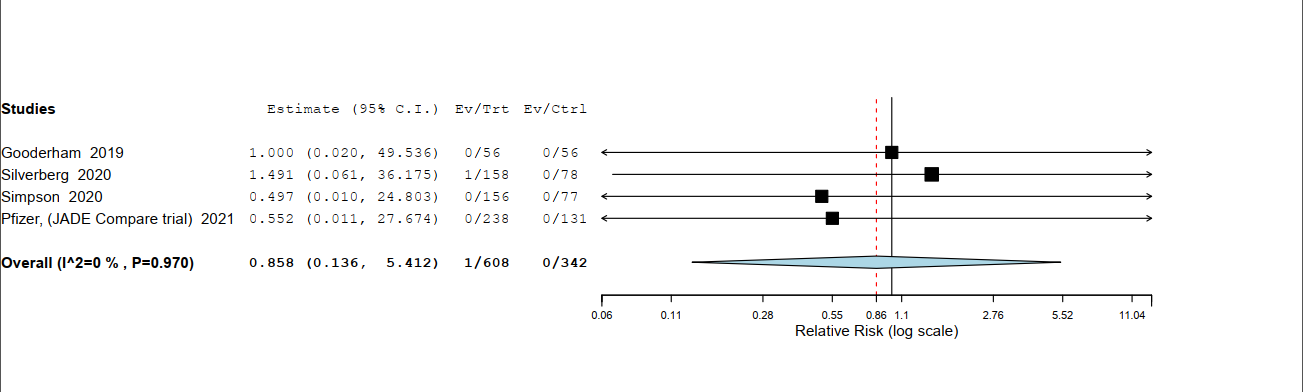
**

**Figure S16**

**
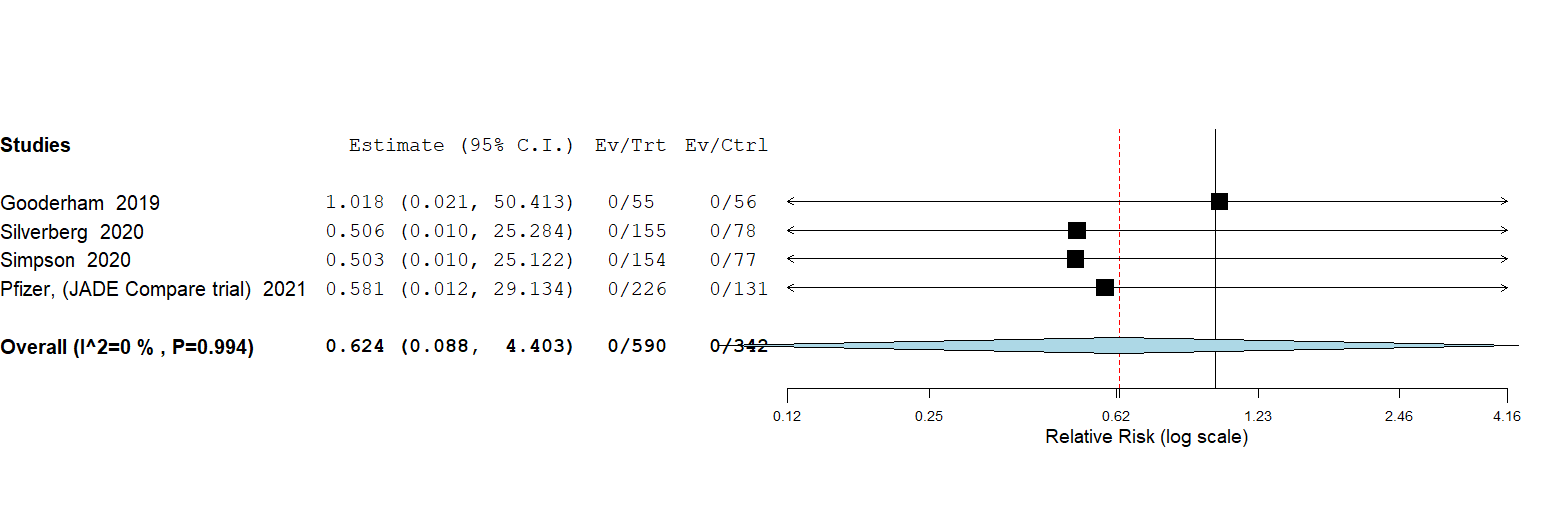
**

**Figure S17**

**
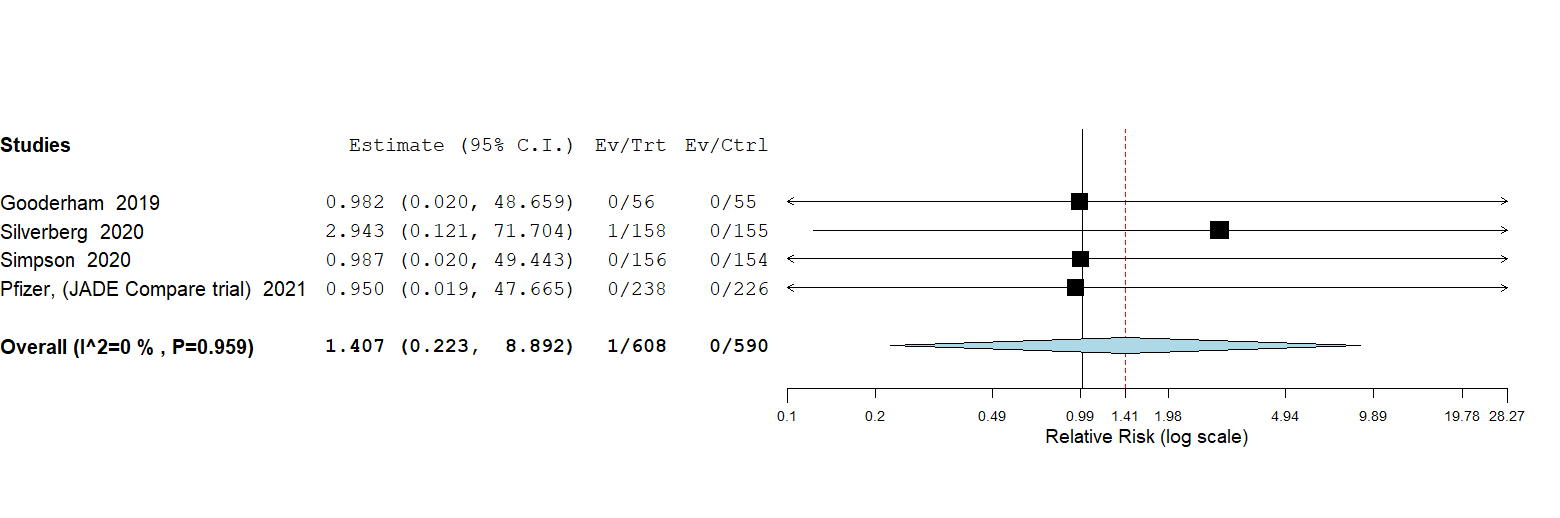
**
